# *In vivo* biomechanical assessment of iridial deformations and muscle contractions in human eyes

**DOI:** 10.1098/rsif.2022.0108

**Published:** 2022-07-06

**Authors:** Babak N. Safa, Mohammad Reza Bahrani Fard, C. Ross Ethier

**Affiliations:** Wallace H. Coulter Department of Biomedical Engineering, Georgia Institute of Technology/Emory University, Petit Biotechnology Building (IBB), 315 Ferst Drive, Room 2306, Atlanta, GA 30332-0363, USA

**Keywords:** iris, pupil, glaucoma, digital image correlation, finite-element method

## Abstract

The iris is a muscular organ whose deformations can cause primary angle-closure glaucoma (PACG), a leading cause of blindness. PACG risk assessment does not consider iridial biomechanical factors, despite their expected influence on iris deformations. Here, we exploited an existing biometric dataset consisting of near-infrared movies acquired during the pupillary light reflex (PLR) as a unique resource to study iris biomechanics. The PLR caused significant (greater than 100%) and essentially spatially uniform radial strains in the iris *in vivo*, consistent with previous findings. Inverse finite-element modelling showed that sphincter muscle tractions were *ca* fivefold greater than iridial stroma stiffness (range 4- to 13-fold, depending on sphincter muscle size). This muscle traction is greater than has been previously estimated, which may be due to methodological differences and/or to different patient populations in our study (European descent) versus previous studies (Asian); the latter possibility is of particular interest due to differential incidence rates of PACG in these populations. Our methodology is fast and inexpensive and may be a useful tool in understanding biomechanical factors contributing to PACG.

## 1. Introduction

**Figure 1.**
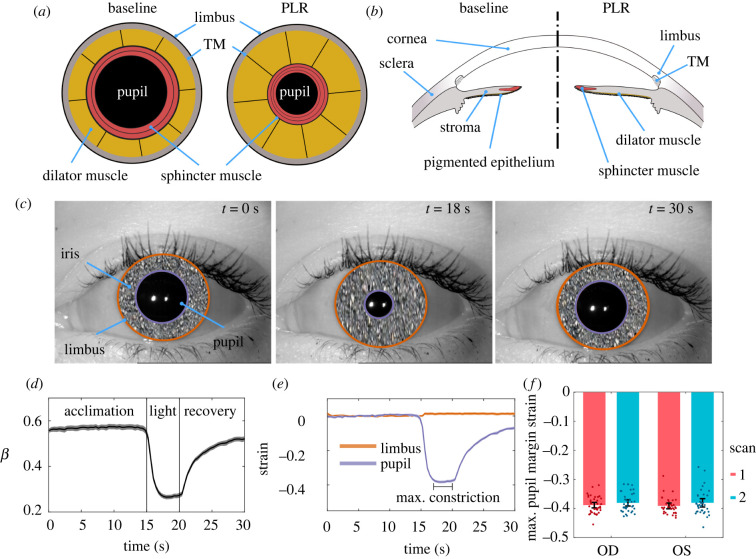
Schematic illustration of the mechanics of pupillary light reflex (PLR) in (*a*) frontal and (*b*) sagittal views, showing the anterior chamber of the eye, including the pupil, the iris, and its attachment to the limbus and trabecular meshwork (TM). When the circumferential sphincter smooth muscle is activated, the pupil constricts (i.e. PLR). (*c*) Three representative images of the PLR from the same subject at the beginning of the test (*t* = 0 s), during maximum constriction (*t* = 18 s), and at the end of recovery (*t* = 30 s). We have obscured iridial surface features to protect the identity of the subject. (*d*) The ratio of the pupil radius to limbal radius ($\beta =r_{p}/r_{l}$; mean as solid line and 95% confidence interval [CI] as shaded area). Initially, the pupil accounted for 56.6% ± 7.0% (mean ± s.d.) of the iris diameter (limbus edge diameter), while at maximum pupil constriction, it reduced to 26.2% ± 4.3%. For purposes of these calculations, we averaged the test–retest measurements for each eye. (*e*) Throughout the PLR test, the limbus diameter did not change and had a negligible strain (1.3% ± 4.3%; single-group *t*-test compared with zero *p* = 0.007). After light exposure, the pupil demonstrated a dramatic 38.6% ± 3.1% (single-group *t*-test compared with zero *p* < 0.001) compressive strain. The graph shows mean and 95% CI over all subjects (shaded area, difficult to distinguish because it is small). (*f*) The tested subjects' peak pupillary margin strain at PLR was not different between the left (OS) or right (OD) eyes, and the results were repeatable between scans. Here, individual data points are shown overlaid with the error bars indicating 95% CI.

The human iris is an annular tissue disc with remarkable properties, including extreme contractility, e.g. iridial contraction can cause pupil diameter to change from 1 to 9 mm in a fraction of a second [1]. Furthermore, the iris's contractions and its anatomical placement in the anterior chamber (figure 1*a,b*) involve the iris in glaucoma, the leading cause of irreversible blindness worldwide [2]. Specifically, in the common form of glaucoma known as primary angle-closure glaucoma (PACG), the iris impedes aqueous humor drainage from the eye, drastically elevating intraocular pressure (IOP) and leading to a potentially blinding medical emergency [3].

Risk factors for PACG include anatomical deficits (e.g. a crowded anterior chamber), age and genetic background [3]; however, these factors alone cannot predict PACG incidence. For instance, a 5-year risk assessment study on an Indian population showed that only 22% of primary angle-closure suspects developed primary angle-closure (PAC; defined as more than 180° angle occlusion with no evident damage to the optic disc and visual field), and none of the PAC cases progressed to PACG within the 5-year period of the study [4]. Similarly, the large ZAP trial [5] showed that only a small percentage of people classified as high risk (PAC suspects) developed PACG within 6 years. In short, the poor predictive power of the existing risk assessment criteria indicates that currently accepted risk factors are incomplete and inadequate.

Iris biomechanics, which strongly influences iridial deformations, is likely to be an additional risk factor for PACG. For example, dilation of the pupil induces a concave curvature of the iris favourable for developing PACG [6]. Importantly, patients with a history of PACG tend to have an iris with higher stiffness and lower permeability [7,8], suggesting clinical utility in the knowledge of *in vivo* iridial biomechanical properties. However, specific biomechanical risk metrics for PACG remain unknown, in part due to the difficulty of characterizing *in vivo* mechanical proprieties of the iris.

Although the iris is optically accessible, its structure is complex, posing challenges to understanding its biomechanics and structure–function relationships. Notably, iridial contractions are driven by two antagonistic smooth muscles, i.e. the sphincter and dilator muscles (figure 1*a*,*b*). Their contractions change iridial morphology (e.g. iris volume [9]), mechanical properties (e.g. stiffness [10] and permeability [11]).

Here, we evaluated the *in vivo* biomechanics of the iris, exploiting the fact that iridial deformation is of interest in a wide range of scientific and technological applications [12]. Specifically, because iris surface features are unique to each individual and are stable throughout life [13], iris recognition is widely used in biometric identification and gaze position estimation in video-based eye-tracking, which have motivated the development of several analysis techniques and acquisition of large datasets containing movies of human iridial motion during the pupillary light reflex (PLR) [14–16]. We used one such publicly available biometric dataset, consisting of near-infrared (NiR) videos of human irides during PLR, which allowed us to calculate *in vivo* iridial strains and estimate muscle traction. We observed strains of larger than 100% and muscle tractions fivefold greater than iris stromal stiffness. The methodology described herein provides a novel approach for *in vivo* evaluation of iris biomechanics using an accessible imaging modality, thus laying the groundwork for future clinical and functional assessment of iris biomechanics in the pathophysiology of glaucoma.

## 2. Results

### 2.1. Pupil and limbus deformations during pupillary light reflex

The iris is highly sensitive to light, with the pupil constricting in response to an increase in light intensity during the PLR. To biomechanically analyse the iris, we quantified iridial deformations by tracking the limbus and pupil during PLR (figure 1*a–c*). We used digital image segmentation and Daugman's method [13,17] to calculate the limbal and pupillary diameters throughout 30 s videos (*n* = 163 videos from 42 subjects; figure 1*c*) and calculated the ratio of pupillary to limbal radii (*β* = *r_p_*/*r_l_*) and hence the Lagrangian strains (*ε_θθ_*) of the limbus and pupil margins. As expected, the limbus did not appreciably deform during PLR (figure 1*c,d*), and the pupil maintained a constant radius in darkness (dark adaptation during acclimation phase; figure 1*d*). However, the pupil dramatically contracted when the eye was exposed to ambient light (figure 1*d,e*), gradually returning towards baseline after light stimulation ended (figure 1*d,e*). The average *β* during acclimation was 56.6% ± 7.0% (mean ± s.d.), reducing to 26.2% ± 4.3% at maximum constriction (figure 1*d*). The average strain of the limbus margin was negligible (*ε_θθ_* = 1.3% ± 4.3%; *t*_79_ *=* 2.75*, p* *=* 0.007*, d*_Cohen_ *=* 0.3, single-group *t*-test compared with zero), while at maximum pupil constriction the pupillary margin strain was *ε_θθ_* = −38.6% ± 3.1% (*t*_79_ *=* 111.32*, p* *<* 0.001, *d*_Cohen_ = 12.5, single-group *t*-test compared with zero; figure 1*e*).

We next analysed variability in pupillary margin strain between scans for the same eye (test–retest) and between fellow eyes from the same subject. A difference in the PLR between the left (OS) and right (OD) eyes is known as a relative afferent pupillary defect (RAPD) and can indicate an underlying medical condition [18]. However, we saw no evidence of RAPD in the 42 pairs of eyes in the dataset (*F*_144_ = 0.029, *β*_LME_ [95% CI] = 0.000 [−0.011, 0.012], *p* = 0.977, linear mixed-effects model (LME)), and the test–retest paradigm did not result in different PLR responses (*F*_144_ = 2.294, *β*_LME_ [95% CI] = 0.008 [0.001, 0.015], *p* = 0.023; figure 1*f*). Although each eye's test–retest scans indicated that the pupillary margin strain was slightly smaller in the second scan, the size of this effect was small, with less than 1% strain difference (0.8% ± 2.9%), which indicates that the PLR provides repeatable metrics.

### 2.2. Spatial distribution of mechanical strain in the iris

**Figure 2.**
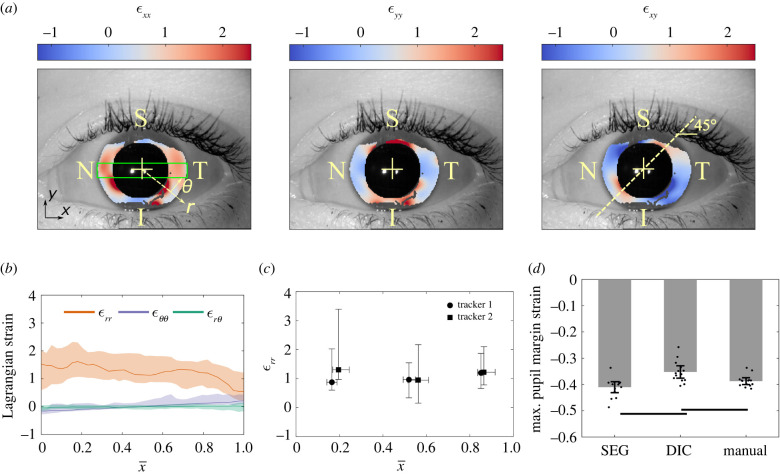
(*a*) Representative in-plane iridial Lagrangian strain field determined using digital image correlation (DIC). The colours indicate the strain at maximum pupillary constriction in the reference configuration. The strain fields demonstrate a symmetrical deformation, i.e. *ε_xx_* is essentially symmetric about the *x*-axis, *ε_yy_* is symmetric about the *y*-axis and *ε_xy_* is diagonally antisymmetric. The colour bar spans 95% of the CI of the data. S, N, I and T denote superior, nasal, inferior and temporal, respectively. (*b*) The spatial distribution of in-plane iridial strain components in a normalized coordinate system. The median and interquartile range (IQR; shaded areas) are shown for the ROI (green box in panel *a* left, with height equal to one-half of the pupil radius during the acclimation phase, and width equal to the limbus diameter), where $\bar{x}=0$ for the pupillary margin, and $\bar{x}=1$ for the limbus. It is evident that there are significant deformations over the entire iris; for example, *ε_rr_* is 1.53 [0.59, 2.01] (median and IQR) at the pupillary margin. The median value of *ε_rr_* is essentially constant across much of the iris and then decreases to 0.54 [0.21, 1.22] at the limbus. As expected, *ε_θθ_* and $\epsilon_{r\theta }$ were small compared with $\epsilon_{rr}$. $\epsilon_{\theta \theta }$ was negative at the pupillary margin (indicating sphincter constriction), and due to the symmetry of deformation, $\epsilon_{r\theta }$ was essentially zero. (*c*) We validated the DIC results by having two trackers annotate structural features manually to calculate *ε_rr_*. By comparing the medians and IQR of *ε_rr_*, it is evident that both trackers acquired similar results compared with DIC. In addition, the results of the two trackers were not different from each other. The vertical and horizontal error bars indicate IQR. (*d*) To further validate the DIC results, we measured the pupil strain by calculating the average pupil margin strain at maximum constriction (manual) and compared it with pupil margin strain results from DIC analyses (DIC) and Daugman's method (SEG). Results obtained by the three methods showed reasonable agreement, with the maximum difference of approximately 15% occurring between DIC and SEG (*p* < 0.01). Error bars indicate 95% CI. The horizontal bars indicate *p* < 0.05/3.

Although deformation at the pupillary margin is of interest, more information can be obtained by determining local deformation across the iris stroma. We therefore performed digital image correlation (DIC; [19]) and calculated components of the iridial Lagrangian strain tensor at maximum pupillary constriction across the iris (figure 2*a*). We observed strain patterns similar to that in an annular disc under axisymmetric radial contraction, with the *ε_xx_* strain component distributed symmetrically about the nasal-temporal (N-T) axis (*x*-axis) and *ε_yy_* being symmetric about the superior–inferior axis (*y*-axis). The in-plane shear strain (*ε_xy_*) demonstrated an antisymmetric distribution across both *x* and *y* axes, with a 45° inclination (figure 2*a*)

We calculated the median of each strain component in the ROI (green box in figure 2*a*) as a function of radial distance from the pupillary margin (see electronic supplementary material, figure S1), where *ε_xx_* is essentially equivalent to *ε_rr_*, *ε_yy_* to *ε_θθ_*, and *ε_xy_* to *ε_rθ_* (figure 2*a*). Plotting these strain components versus normalized distance ($\bar{x}$) from the pupillary edge, we found that *ε_rr_* was 1.53 [0.59, 2.01] (median and [interquartile range (IQR)]) at the pupillary edge and was almost uniform across the iris, with a localized decline to 0.54 [0.21, 1.22] close to the limbus (figure 2*b*). In addition, both *ε_θθ_* and *ε_rθ_* were small compared with *ε_rr_*. *ε_θθ_* was negative at the pupillary margin, consistent with sphincter constriction, and as expected due to symmetry in the iris deformation, *ε_rθ_* was almost zero (figure 2*b*).

To validate the DIC results, we compared them with strains obtained from two manual annotations, one for the spatial distribution of radial strain and the other for pupillary margin strain. First, two independent annotators manually tracked iridial features along the N-T axis (figure 2*a*), from which we calculated the radial Lagrangian strain, *ε_rr_*, at maximum pupil constriction. The manual tracking results agreed with the DIC results, as demonstrated by comparing the median and IQR of the strains (figure 2*b,c*). Further, the results of manual feature tracking were not different between the annotators (*F*_1,82_ < 0.001, *p* = 0.981, two-way ANOVA), and the comparison between iris regions did not indicate a difference (*F*_2,82_ = 0.896, *p* = 0.413, two-way ANOVA). No interaction effect was detected between annotators and regions (*F*_2,82_ = 0.229, *p* = 0.796, two-way ANOVA).

We next manually calculated the maximum pupillary margin strain based on the change in the average diameter of the pupil during maximum constriction, calculated by averaging the diameter of the pupil along the N-T and superior–inferior axes (see electronic supplementary material, figure S2B). We compared these results with pupillary margin strain measured from DIC and segmentation/Daugman's method (described above). The values of pupillary margin strain were generally consistent across the methods (figure 2*d*), albeit with different quantitative results between methods (*F*_2,41_ = 13.324, *p* < 0.001, two-way ANOVA), which was not dependent on the scan (*F*_13,41_ = 1.887, *p* = 0.082, two-way ANOVA). Only the strains from DIC showed a difference from the segmentation-based strains (Δ*ε_p_* = 16.4%; *t*_13_ = 3.929, *p* = 0.002, *d*_Cohen_ = 1.5, paired *t*-test) and manual pupillary strains (Δ*ε_p_* = 9.0%; *t*_13_ = 3.827, *p* = 0.002, *d*_Cohen_ = 1.1, paired *t*-test), while the segmentation-based versus manual-based strain difference was not significant (Δ*ε_p_* = 5.9%; *t*_13_ = 2.533, *p* = 0.025, *d*_Cohen_ = 0.8, paired *t*-test), i.e. greater than the Bonferroni-corrected significance level of 0.05/3.

### 2.3. *In vivo* assessment of sphincter muscle traction

**Figure 3.**
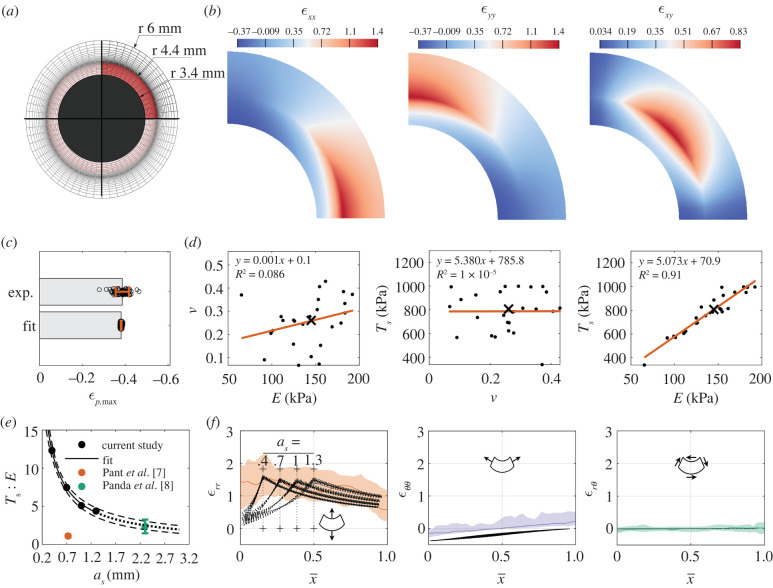
(*a*) The finite-element (FE) mesh (sphincter width $a_{s}=1\;\mathrm{mm}$) used to model iridial biomechanics. The active sphincter muscle is highlighted in red. (*b*) An example of the strain field obtained from FE simulations, where we leveraged the symmetry of the iris for numerical efficiency to model only a one-eighth wedge of the entire iris, i.e. half the iris thickness and a one-quarter sector. (*c*) The model fitted the median experimental maximum pupillary strain very well ($a_{s}=1\;\mathrm{mm})$. Error bars are mean ± s.d. (*d*) Cross-plots between the fitted model parameters, *E*, ν and *T_s_*, ($a_{s}=1\;\mathrm{mm}$, individual fit parameter values as solid dots, and the median as ‘×’) showing that the model fitting was insensitive to a change in ν, and interestingly that there was a strong linear correlation between *E* and *T_s_*, with the latter being approximately five times the former. The linear regression results are shown in each panel, where ‘*y*’ corresponds to the parameter in the vertical axis and ‘*x*’ to the horizontal axis. Note that the individual points correspond to the outputs of multi-start optimization and do not correspond to each subject. (*e*) Repeating the data-fitting using different *a_s_* showed a nonlinear inverse correlation between the *T_s_* : *E* ratio and *a_s_*. The dashed-line indicates the 95% CI of the nonlinear regression analysis. The dotted-line indicates extrapolation of the model. The coloured data points indicate calculated values from the literature shown as mean ± s.d. (*f*) Spatial distributions of Lagrangian strains (in polar coordinates) versus normalized position across the iris, where dashed lines show the results from the FE models for different *a_s_*; coloured lines and shaded regions show DIC measurements and 95% CI (repeated from figure 2*b*). The model predictions and the experiments were in general agreement, especially when comparing the peak radial strains with the median experimental strains in the body of the iris; however, the shape of the radial strain was sensitive to *a_s_*, where the peak radial strain occurred at the edge of the sphincter muscle (identified by vertical dotted lines and ‘+’ signs).

Next, we used experimentally measured pupillary margin strains to evaluate iridial biomechanical properties *in vivo*. We modelled the iris using an eightfold symmetric finite-element (FE) mesh, with the inner pupillary elements representing the sphincter muscle (sphincter width *a_s_* = 1 mm; figure 3*a,b*) [20,21]. We performed multi-start data-fitting [22], using the measured mean maximum pupil margin strain of all the subjects as the target value and the model parameters being stromal modulus *E* (kPa), Poisson's ratio *ν* and sphincter muscle traction *T_s_* (kPa). Interestingly, it was evident that the model fits were not sensitive to *ν*, and that there was a linear correlation between *E* and *T_s_*, with *T_s_* : *E* ≈ 5 (figure 3*d*). The *T_s_* : *E* ratio is important as it provides a basis for objective assessment of iris biomechanics from pupillary size changes, as discussed below. Unfortunately, due to our data's two-dimensional nature, we could not uniquely identify a Poisson's ratio for the iris (figure 3*d*).

The force exerted by a muscle is dependent on its dimensions [23]; therefore, we also conducted the above data-fitting while varying sphincter muscle width over a physiological range *a_s_* = [0.4 mm, 0.7 mm, 1.3 mm]. We observed that increasing *a_s_* caused a decrease in the traction (*T_s_* : *E* ratio) needed to achieve the same pupillary strain, from *ca* 13 to 4. The relation between *T_s_* : *E* and *a_s_* was nonlinear and could be fit by the following empirical relation:

2.1 $$T_{s}\;:\;E={\left(A/a_{s}\right)}^{B},$$

where *T_s_* : *E* is non-dimensional, *a_s_* is in mm, *A* = 6.197 [3.723, 8.671] mm (mean [95% confidence interval] and *B* = 0.916 [0.768, 1.064] is non-dimensional (figure 3*e*).

A further effect of changing *a_s_* was alteration in the spatial distribution of iridial strains. The simulated strain responses for different *a_s_* values were compared with each other and to the values experimentally measured using DIC, as described above (figure 2*b*). We observed that changing *a_s_* affected the spatial distribution of the radial strain (*ε_rr_*); however, it did not change the spatial distribution of *ε_θθ_* and *ε_rθ_* (figure 3*f*), despite the simulations having different sets of material parameter values. Further, the peak *ε_rr_* was the same for all the models and agreed with the DIC results. However, within the sphincter muscle, *ε_rr_* had a different distribution compared with the experimentally measured strains, with the model having a smaller *ε_rr_* value compared with the experimental data. There was agreement between the experimental and modelled *ε_θθ_* and *ε_rθ_*, albeit with a negative shift in *ε_θθ_* of the models compared with the experimental values. Specifically, the model's predicted *ε_θθ_* was *ca* −40%, which is consistent with the strain determined from changes in pupillary diameter, suggesting that the DIC underestimated *ε_θθ_*.

## 3. Discussion

The iris plays a central role in PACG. Worldwide, PACG is the second most prevalent form of glaucoma, although in some regions, primarily in parts of East Asia, PACG is the most prevalent form [24]. Current risk assessment in PACG patients is based on precise anatomical measurements of the anterior chamber and iris, e.g., by optical coherence tomographic (OCT) imaging [3,25], yet the predictive power of such techniques is poor [5]. This motivates the development of novel techniques for identification and assessment of PACG risk factors.

Iris biomechanical properties have been largely ignored as potential risk factors for PACG despite their likely importance. There are two challenges in incorporating iris biomechanics into clinical management of PACG. First, knowledge about iris biomechanical properties is scarce. Second, there are currently no clinical techniques to measure iris biomechanics. Ideally, such techniques would be inexpensive, i.e. suitable for patients in less economically developed settings. Here, we repurposed a publicly available biometrics dataset to track the deformations of the iris during the PLR and hence analyse the *in vivo* biomechanical properties of the human iris. Inducing and measuring PLR is accessible and reproducible, and thus our approach is amenable to future translational studies of iris biomechanics.

Our data showed that only by using pupil margin strain, the sphincter muscle traction (*T_s_*) and iris stroma's stiffness (*E*) could not be uniquely identified; however, quite interestingly, they were linearly correlated, with a mean *T_s_* : *E* ratio of 5 (range 4–13, depending on sphincter muscle width). We note that the *Warsaw-BioBase-Pupil-Dynamics v3* dataset [26] does not include information about the glaucoma status of subjects; therefore, it is possible that some glaucomatous subjects were inadvertently included in this dataset. Nonetheless, the correlation between *T_s_* and *E* is consistent with previous studies, where in non-glaucomatous human subjects, Pant *et al.* [7] estimated *T_s_* : *E* = 1.08 ± 0.16, and Panda *et al.* estimated *T_s_* : *E* = 2.34 ± 0.90 [8] (figure 3*e*), while in subjects with a history of PACG, the sphincter muscle was determined to be relatively weaker, with *T_s_* : *E* = 0.38 ± 0.10 [7] and *T_s_* : *E* = 1.63 ± 0.56 [8].

It is of interest to note that the *T_s_* : *E* values from previous studies are notably smaller than our estimate of *T_s_* : *E* (figure 3*e*). There are multiple interrelated factors that probably influence this difference, as follows.

— *Sphincter muscle size*. Panda *et al.* used a sphincter muscle size much larger than ours. Their muscle size was obtained based on measurments in porcine eyes, yet there are notable anatomic differences between pig and human irides (e.g. elliptical pupils), suggesting an over-estimation of human muscle size in their study. Interestingly, our empirical relation (equation (2.1)) is consistent with the *T_s_* : *E* value that they report (figure 3*e*), i.e. if Panda *et al.* had used smaller sphincter width in their study, they may have arrived at a similar *T_s_* : *E* ratio as us. Unfortunately, there are currently no *in vivo* methods for determining sphincter muscle dimensions, motivating the development of techniques (e.g. feature tracking and correlating sphincter size with local iris deformations [15]) for assessing sphincter muscle size in human subjects.
— *Methodological differences*: Pant *et al.* found a *T_s_* : *E* ratio threefold smaller than ours, even though the sphincter muscle width that they used (approx. 0.73 mm) lay within our range (*a_s_* = 0.4–1.3 mm). However, they used less extreme lighting conditions to induce PLR, resulting in smaller iridial radial strains than we observed (approx. 12% versus 100%). Presumably, this means that the sphincter muscle was not maximally stimulated in their study, emphasizing the importance of methodological details.
— *Genetic background:* Pant *et al.* studied an Indian population, and Panda *et al.* studied a Singaporean one (Indian/Chinese ethnicity), while our data originated in Poland. Although patient demographics were not available for our population, it is highly likely that subjects were of European descent. We speculate that populations of European descent have a larger *T_s_* : *E* than Asian populations, due to several related observations. First, PACG is more prevalent in Asia, including both India and Singapore, than in the rest of the world [24]. Second, PACG patients have stiffer irides [7,8]. Clearly, further study is required to evaluate whether there are differences in iridial biomechanics between different populations. Identification of such differences would complement established anatomical risk factors in genetically diverse clinical populations.

We showed that the iris experiences radial strains (*ε_rr_*) of greater than 100% during PLR (figure 2), which is consistent with previous reports using manual feature tracking [12,27]. Further, we observed that the circumferential strain (*ε_θθ_*) was also significant (figure 2*a,b*). At the pupil edge, *ε_θθ_* calculated from the FE model matched *ε_θθ_* computed from tracking pupil diameter experimentally, but not values of *ε_θθ_* measured by DIC (figure 3*f*). In general, although the iris margin strains were accurate (figure 1*d–f*), the DIC had less reliable results closer to the pupillary edge while providing more deformation information across the iris. This lower reliability of DIC near the pupillary margin was also evident from the low estimation of pupillary margin strain by DIC, with as much as 10–15% difference from SEG and manual calculation (figure 2*d*). Probably this discrepancy is due to the radial orientation of iris surface features and the large deformations in the radial direction, complicating DIC imaging. It is possible that using alternative approaches to evaluate the local strains in the iris (e.g. feature tracking [15]), could be useful for better evaluating iridial strains. Nevertheless, higher resolution imaging would be needed to more accurately calculate the circumferential strains of the iris by DIC.

This study was subject to several limitations. For example, we did not include the dilator muscle in our analyses. However, the effects of dilator muscle traction during PLR are minimal [28]. Additionally, since we used two-dimensional images, there were potential confounding factors due to the curvature of the iris, distortions due to corneal refraction, and reflected light on the cornea. For example, we observed a subtle decline in the median radial strain near the limbus (figure 2*b*), potentially due to distortion due to corneal refraction in this region. In addition, corneal reflections added noise which complicated feature tracking and DIC (see electronic supplementary material, figure S4). Future studies could benefit from using three-dimensional imaging modalities (e.g. optical coherence tomography (OCT) [29]) and elastography techniques (e.g. optical coherence elastography (OCE) [30,31]) to complement the NiR imaging-based technique presented herein. Further, due to the two-dimensional nature of our data, our model could not uniquely evaluate the iridial Poisson's ratio. Previous studies have shown that using three-dimensional imaging and three-dimensional FE modelling of the iris could also be helpful to identify additional *in vivo* mechanical properties, such as anisotropic material properties and Poisson's ratio [32]. Such a three-dimensional modelling scheme could also be useful in creating a more physiologically accurate model of the iris by considering its varying thickness, curvature, and spatial distribution of the sphincter muscle thickness, which were not considered in our simplified semi-two-dimensional FEM model. Finally, we did not consider viscoelastic and nonlinear stromal biomechanical behaviours, which would be worth adding to the model to better replicate iris physiological behaviour [8,33].

In conclusion, we measured iridial deformations and determined tissue mechanical properties *in vivo* using imaging of the PLR and FE modelling. Our technique for measuring iris biomechanics is simple and does not require specialized devices, and therefore has significant potential for clinical translation. This study establishes proof-of-concept for using pupillography during the PLR to functionally assess iris biomechanics *in vivo*, of interest in evaluating iris biomechanics' role in glaucoma.

## 4. Material and Methods

### 4.1. Pupillary light reflex dataset

To assess the tissue deformations induced by the activation of the iris sphincter muscle, we used the publicly available *Warsaw-BioBase-Pupil-Dynamics v3* dataset [26], which includes 163 videos (each 30 s long, acquired at 25 Hz) of PLR from 42 subjects of ages 20–50 years. The images had an approximate resolution of 39 µm pixel^−1^, which we calculated based on the typical limbus diameter of 12 mm [34] and the average diameter of the limbus in pixels. Each eye scan video has a unique code; e.g. *10066left2* denotes the second scan of subject 10066's left eye. To obtain scans, the subject's head was placed in a large shaded box to prevent penetration of ambient light, and built-in LEDs were used to induce the PLR. Images were acquired in the NiR using a custom system (IrisCube [35]). NiR imaging is standard practice in pupillography [36], where light with a wavelength less than 800 nm is absent, allowing imaging in darkness and detection of pupillary reflexes independent of the stimulus lighting. Both the left (OS) and right (OD) eyes of subjects were scanned twice. Scans included a 15 s acclimation phase in the dark (dark adaptation), a 5 s exposure of the eye to LED light, followed by 10 s of darkness (figure 1*a*). The 5 s exposure to visible light induced pupillary constriction and the elimination of this stimulus allowed for partial pupil recovery. We note that full pupil size recovery can be achieved with a longer period of darkness after the light stimulus [37,38]; fortunately, the lack of full recovery in this dataset did not affect our analysis, since we were only interested in maximum pupil constriction.

### 4.2. Pupil and limbus segmentation and deformation

To assess the deformation of the pupil and limbus, we used an automated algorithm. Given the enormous volume of data, we analysed every 10th image in the videos, resulting in an effective 2.5 Hz frequency, equivalent to 400 ms temporal resolution. Unless otherwise specified, all analyses were performed in Matlab. Specifically, to measure pupil edge diameter, we used a custom pixel intensity-based threshold segmentation of the pupil, where we first applied a median filter (*medfilt2()* function; window size = [3 pixel × 3 pixel]) to reduce image noise, followed with a binarization function based on Otsu's method (*imbinarize()* function) with a 0.1 threshold. Next, to obtain a final pupil mask, we performed an erosion and dilation routine (*imerode()* and *imdilate()* functions) with a 2 pixels-wide square morphological element (*strel()* function), and fill hole (*imfill()* function). We then calculated the average pupil radius as $r_{p}=\sqrt{\mathrm{area}/\pi }$. To measure limbus radius, we used a publicly available implementation of Daugman's method in Matlab [13,26]. The outputs of this step were the fitted radii of the pupil and limbus. We calculated the ratio of the pupillary (*p*) to limbal (*l*) radii as

4.1 $$\beta =\frac{r_{p}}{r_{l}}.$$

We also calculated the Lagrangian strain of both the pupillary margin and limbus as

4.2 $$\epsilon_{i}=\frac{1}{2}\left[{\left(\frac{r_{i}}{{\bar{r}}_{i0}}\right)}^{2}-1\right],$$

where ${\bar{r}}_{i0}$ is the average value of *r_i_* over the initial 15 s acclimation phase (figure 1*c*), and *i* = *p* for pupil and *i* = *l* for limbus.

We calculated the maximum pupillary margin strain, used in the FE analysis, as the mean strain over the interval 17–20 s. We averaged the test–retest scans for each eye, and then calculated the mean, standard deviation and 95% confidence interval of the maximum pupillary margin strain for the entire dataset. As quality control, we identified failed segmentations by performing a *post hoc* outlier identification, where the segmentations having maximum pupillary margin strain values more than three times the standard deviation away from the mean were excluded from the analysis, so the final count of successful segmentations was 147. To test whether repeated scans of each eye or the eye's anatomical placement (OS/OD) affected the PLR, we used an LME with the pupillary margin strain as the observed parameter, fixed effects being the order of scan (scan 1 and scan 2) and anatomical placement (OS and OD), and random effect being the anatomical placement grouped based on subject (significance level *α* = 0.05).

### 4.3. Spatial distribution of strain and digital image correlation

We calculated the deformation in the iris stroma during PLR using DIC. We conducted the DIC analysis using Vic2D software (Correlated Solution, Irmo, SC, USA) on *n* = 17 videos from nine unique eyes from seven subjects. Some of the analysed videos were repeat scans of the same eye; however, due to the randomness of the gaze, blinking and corneal reflection patterns, we treated each video as an independent sample for the DIC analysis.

The images (768 pixels wide × 576 pixels high) were loaded into Vic2D using the tagged image file format (tiff). We conducted an incremental correlation (subset size of 31 pixels and step size of 4) on a manually traced reference ROI around the iris that excluded the pupil and eyelids from the analysis. We used normalized sum square difference (NSSD) and correlation function, where the matchability threshold was set at 0.1 pixels, and the Lagrangian strain was calculated in a post-processing step with a filter size of 15 pixels. We analysed the images after the beginning of light stimulation, i.e. during pupillary constriction. To avoid the effect of blinking, which could terminate the DIC tracking, we manually excluded images in which blinking occurred while maintaining the time label of each image. For consistency, we used the same protocol for all DIC analyses.

We evaluated the spatial distribution of the Lagrangian normal and shear strains along the N-T axis at maximum pupillary constriction. The strain fields near the superior and inferior regions were not reliable due to coverage by the eyelids (see electronic supplementary material, figure S3). We evaluated the strain along the N-T axis by calculating the median of the strains along the superior–inferior axis in a rectangular ROI that passed through the pupillary centre, of height one-quarter of the pupillary diameter and width equal to the limbus diameter (green box in figure 2*a*; electronic supplementary material, figure S1). To maintain a consistent coordinate system for all the strain fields, we used a normalized distance from the pupil margin in which the pupillary margin had a coordinate value of zero ($\bar{x}=0$), and the limbus had a value of 1 ($\bar{x}=1$). We conducted a *post hoc* outlier identification analysis based on the Hausdorff distance [39] of the strain component curve versus $\bar{x}$ and excluded four videos from the DIC analysis; however, this had a minimal effect on the results (see electronic supplementary material, figure S5).

To validate the DIC results, we used two procedures. First, two separate annotators (trackers) manually tracked eight points along the N-T axis using ImageJ [40] in a subset of the videos analysed by DIC (*n* = 8). Due to the labour-intensive nature of manual point tracking, we only used two annotators; however, using more annotators could possibly produce more accurate results. Tracker 1 first carried out the DIC analysis, and then annotated the images. Ideally the annotations of Tracker 1 would have been masked; however, because strains are computed from spatial derivatives of locations, the DIC information is not expected to affect manual feature tracking. Tracker 2 independently annotated the images while masked to the results of Tracker 1 and the DIC analysis. We selected approximately equally distanced points along the N-T axis to divide the area between limbus and pupil into three roughly equal parts (see electronic supplementary material, figure S2). However, the choice of points was limited by the traceability of features with unaided human vision. We calculated the Lagrangian strain along the N-T axis at maximum pupillary constriction and conducted a two-way ANOVA, where the factors were trackers, regions and their interaction (*α* = 0.05). Second, we compared the pupillary margin strains measured from segmentation (*ε_p_*_,max_ SEG) with the pupillary margin strain measured using a virtual tensometer in Vic2D (*ε_p_*_,max_ DIC). For the latter comparison, we also added another set of manual measurements of pupillary strain at maximum constriction (*ε_p_*_,max_ manual) (*n* = 17), where we calculated the average of the Lagrangian pupillary margin strain at three time-points (frames 425/750, 463/750, 500/750) according to equation (4.2), with the onset of light stimulation (frame 375/750) being the reference (see electronic supplementary material, figure S2). Next, we conducted an additive two-way ANOVA with the factors being analysis method (SEG, DIC and manual) and the identification code of each eye scan (*α* = 0.05), followed by a *post hoc* paired *t*-test with Bonferroni correction (*α* = 0.05/3), where we also report Cohen's effect size (*d*_Cohen_). The pupillary margin strain could not be calculated for three movies because the DIC algorithm failed to pass internal quality control thresholds at the pupillary margin; therefore, we excluded those samples from the ANOVA.

### 4.4. Finite-element modelling of the iris

We created an idealized semi-two-dimensional model of the iris, composed of an eightfold symmetric portion of a disc under plane-stress boundary conditions, motivated by the assumption that anterior and posterior chambers were at the same pressure, resulting in zero net force loading. Details of the model boundary conditions are shown in electronic supplementary material, figure S6. We took the iris during the acclimation phase (figure 1*d*) as the reference state. The outer radius of the model was 6 mm [34], and the thickness of the model was 0.17 mm (based on average iris thickness of 0.34 mm [41]). We set the inner radius of the model (pupillary radius) to 3.4 mm, which was calculated based on the outer radius of the iris and the average ratio of the pupillary and limbal radii during the acclimation period (*β*, figure 1*c*). We used 2250 hexahedral elements (HEX8) to generate the mesh, based on a preliminary mesh density sensitivity analysis.

We modelled the iris’s mechanical response using a hyperelastic stromal substance with embedded uniaxial active traction elements to represent the sphincter muscle (figure 3*a*). To simplify the model, we assumed that the sphincter was distributed across the radius in a ring of thickness *a_s_* = 0.4 – 1.3 mm [19,20]. By considering normal PLR function, where light triggers the autonomic nervous system to actuate the sphincter muscle, we modelled the sphincter muscle as a one-dimensional active material along the periphery of the pupil edge, i.e. the Cauchy stress due to the sphincter muscle was [7]:

4.3 $$T_{s}=J^{-1}T_{s}(r)e_{\theta }⊗e_{\theta }.$$

Here, *T_s_* is the magnitude of the sphincter muscle traction, *r* is the distance from the pupil centre, ***e****_θ_* is the circumferential unit vector along the sphincter muscle in the deformed state and *J* is the Jacobian of the deformation gradient tensor. Here, muscle traction was defined as the muscle contractile force divided by muscle cross-sectional area (normal to the pupil periphery). Further, we described the mechanical response of the stroma using a compressible neo-Hookean constitutive relation

4.4 $$\Psi =\frac{E}{2(1+\nu )}\left[\frac{1}{2}(I_{1}-3)-lnJ\right]+\frac{E\nu }{2(1+\nu )(1-2\nu )}{\left(lnJ\right)}^{2}$$

where *Ψ* is Helmholtz's free energy, *I*_1_ is the first invariant of the Cauchy–Green deformation tensor; *E* is Young's modulus (stiffness) and *ν* is Poisson's ratio. The model was implemented and solved using the FEBio software suite (FEBio v. 3.1 [42]), and an example of the FE model's output is shown in figure 3*b*.

### 4.5. Parameter identification

We used the absolute value of the difference between the experimental and the modelled pupillary margin strain at maximum constriction (taken as the average over the period 17–20 s) to perform data-fitting, with parameters *E*, *ν* and *T_s_*. We used a multi-start optimization method [21] with a grid size of 25, to eliminate bias to one initial guess. We set a wide search space with 0 < *E* < 1000 kPa, 0 < *ν* < 0.5 and 0 < *T_s_* < 1000 kPa, which was informed by values previously reported in the literature [7,8,10,30,43]. We performed the data-fitting based on a baseline sphincter muscle width of 1 mm, and due to the sensitivity of the results to the assumed value of sphincter muscle width, we repeated the simulations for *a_s_* = [0.4 mm, 0.7 mm, 1.3 mm]. Using the resulting fitted values, we nonlinearly regressed the ratio of sphincter traction to stroma stiffness versus *a_s_*.

## Data Availability

The PLR image dataset used in this study is available from its publishers (website: http://zbum.ia.pw.edu.pl/EN/node/46/, email: m.trokielewicz@elka.pw.edu.pl). In addition, FEBio's open-source code is publicly available at https://febio.org/. Finally, all of the materials supporting the findings of this study can be accessed at https://doi.org/10.5281/zenodo.6642587. Additional supplementary figures are provided in electronic supplementary material [44].
