# Correction to: ‘*In vivo* biomechanical assessment of iridial deformations and muscle contractions in human eyes’ 2022 by Safa *et al.*

**DOI:** 10.1098/rsif.2022.0788

**Published:** 2022-11-09

**Authors:** Babak N. Safa, Mohammad Reza Bahrani Fard, C. Ross Ethier

*J. R. Soc. Interface* **19**, 20220108. (Published 6 July 2022). (https://doi.org/10.1098/rsif.2022.0108)

The authors regret that they have discovered two errors in the bibliography, which they would like to correct herewith.

1. The citation to reference 17 in §2.1 should be to reference 26, and citations 18–26 in §2.1–4.1 should be to references 17–25, respectively.

2. Reference 41 should read:

Marchini G, Pagliarusco A, Toscano A, Tosi R, Brunelli C, Bonomi L. 1998 Ultrasound biomicroscopic and conventional ultrasonographic study of ocular dimensions in primary angle-closure glaucoma. *Ophthalmology* **105**, 2091–2098. (doi:10.1016/S0161-6420(98)91132-0)

